# Smart therapies against global pandemics: A potential of short peptides

**DOI:** 10.3389/fphar.2022.914467

**Published:** 2022-08-15

**Authors:** Vasso Apostolopoulos, Joanna Bojarska, Jack Feehan, John Matsoukas, Wojciech Wolf

**Affiliations:** ^1^ Institute for Health and Sport, Victoria University, Melbourne, VIC, Australia; ^2^ Immunology Program, Australian Institute for Musculoskeletal Science (AIMSS), Melbourne, VIC, Australia; ^3^ Technical University of Lodz, Department of Chemistry, Institute of General and Ecological Chemistry, Lodz, Poland; ^4^ Department of Physiology and Pharmacology, Cumming School of Medicine, University of Calgary, Calgary, AB, Canada; ^5^ NewDrug, Patras Science Park, Patras, Greece

**Keywords:** pandemics, short peptides, viral and bacterial infections, bioterrorism, smart therapy

## Background

As we entered the third year of this pandemic, since the World Health Organisation (WHO) declared in March 2020 the novel coronavirus severe acute respiratory syndrome (SARS-CoV2) outbreak as a global pandemic COVID-19 (COronaVIrus Disease 19), we are still fighting with newer and newer viral mutations. The pandemic has passed the grim milestone of over 6.4 million COVID19 deaths, from more than 550 million reported cases thus far. In fact, more than 15 million people can die by the end of this year. It is highly likely that this pandemic will become endemic, while the full evolutionary potential of coronaviruses has yet to be revealed. The next pandemic is coming. A microbe with features of SARS-Middle East respiratory syndrome (MERS) and SARS-CoV-2 could lead to significantly more catastrophic loss of life. The co-evolution with other viruses should not be neglected. According to the WHO, we should expect diverse zoonotic, outbreak-prone microbes, including highly pathogenic strains of influenza, Nipah, Ebola, Zika, or hemorrhagic fever viruses. ‘*It’s an evolutionary certainty that there will be another virus with the potential to be more transmittable and deadly than this one*’, said Tedros Adhanom Ghebreyesus, director-general of the WHO. On the other hand, in both poor countries and regions of armed conflict, where vaccination is hampered, historic diseases are re-emerging, with migration and displacement influencing transmission risk and limiting control, and raising potential for additional outbreaks. Furthermore, there are other looming terrible threats to humanity as damaging as the bubonic plagues, such as bioterrorism or antibiotic resistant microorganisms. In most cases, both effective prevention and treatment options are limited.

## Global response to urgent needs: between threat and hope

When at the G20 summit in November 2020 the WHO called for proactive intervention to rising cases, always being on the *qui vive*, the challenge was not only to design a super-jab against dangerous variants of SARS-CoV-2 and other coronaviruses, but also pan-vaccines, or libraries of prototype theranostics against threats from critical and yet unknown pathogens.

**SCHEME 1:**
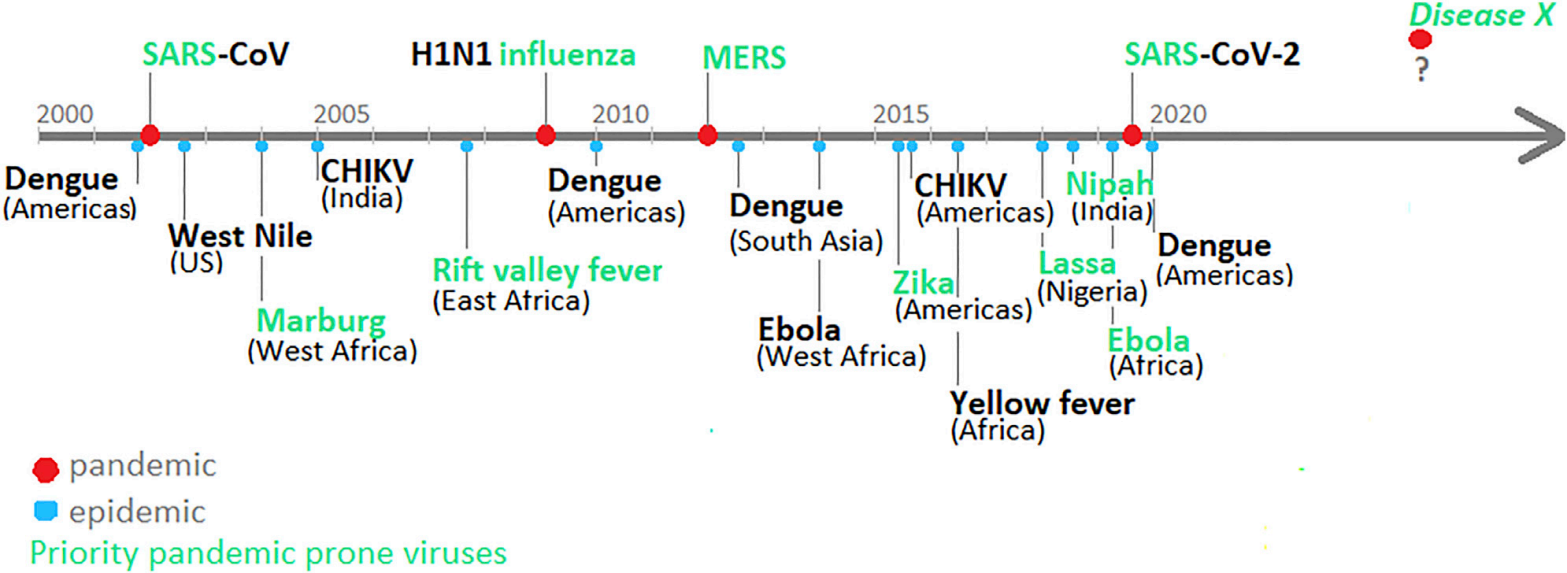
Timeline of XXI century viral pandemics and other outbreaks. Priority pandemic-prone viruses, according to the WHO, in green. (CHIKV- Chikungunya virus).

According to the WHO, the next pandemic can be caused by (re)emerging viruses, their combination or new mutations, or new pathogen (called diseases X). In particular, viruses from Coronaviridae (SARS, MERS), Flaviviridae (West Nile, Zika, Yellow fever, Dengue virus), Togaviridae (Chikungunya virus–CIKV), Arenaviridae (Lassa fever) or Filoviridae (Ebola, Marburg virus) family, that resulted in epidemics and pandemics in the XXI century, are considered as main threat (see Scheme 1). All mentioned zoonotic viruses frequently spillover into livestock and other animals, serving as reservoir hosts for spillover into humans. However, the prediction of the next global outbreak is difficult, beyond recognition of the existence of pre-epidemic forms circulating in reservoirs (Meganck and Baric, 2021).

Moving forward, a little later, one year after the outbreak, the RNA-vaccines, developed at an unprecedented pace, seemed to be helpful in a rapid response to next onslaughts. Nevertheless, they are neither ideal nor a cure-all, especially in the face of quickly evolving and spreading viruses, vaccine diplomacy or ‘apartheid‘. Vaccines take time to either deploy or generate protective immunity. We now know that 70% of world is not be fully vaccinated in mid-2022, while COVID-vaccines will reach poorest countries in 2023. On the other hand, the conventional anti-infectives have inadequate response, insufficient activity, adverse side effects, and an increased rate of resistance (Pour et al., 2019), while the discovery of new drugs is a long and very expensive process. A repurposing strategy was considered as a faster and cheaper option, but many known repurposed drugs failed, while some of them need further thorough studies in relation to relative pathogens (Hanisch and Rake, 2021). What is more, long-term effectiveness and side effects of experimental drugs against SARS-CoV-2 are unknown in detail.

In view of the foregoing, WHO presented a new international treaty on WHO constitution priority to negotiate, and called to strengthen pandemic preventions and preparedness to stop pathogens early and equitably all over the world. Thus, we should rethink directions of actions from a ‘one bug, one drug‘ model to broadly active and more adaptive therapeutic approaches (Dolgin, 2021), which were overlooked this time. We should optimize vaccine platforms to develop next generation pan-vaccines. We should invest in cutting-edge advanced research because innovations save lives. We need smart theranostics before pathogens outsmart us again. It is incredible that in the XXIst century so many diseases are still incurable. We live in smart homes, use smart-phones, build smart-cites, while smart therapies should be our priority to protect our life and health.

Paradoxically, ongoing pandemic has pushed novel biotechnologies at an incredible rate, with a significant progress in diverse fields of science, deepening knowledge on respiratory illnesses, and the ongoing numerous (pre)clinical studies. In consequence, we are entering a new era of revolutionized medicine, with safe and effective smart therapeutic strategies on the horizon.

## Smart strategy

**FIGURE 1:**
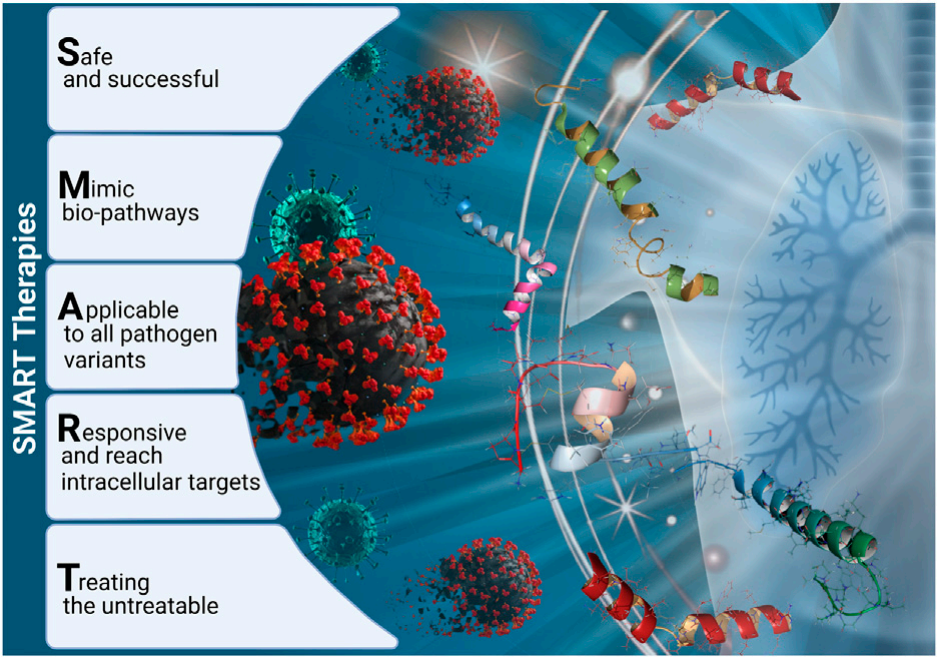
Main features of smart peptide-based therapeutic strategy.

Smart therapy means safe and effective therapy that keeps up with evolving pathogens, closely mimics bio-pathways, with high specificity and selectivity, precision, and flexibility. It can be controllable and sensitive to specific (bio)molecular stimuli, opening the possibility of treating the ‘untreatable‘. Smart therapeutic strategy is based on smart bio-molecules, smart bio-technical solutions against quickly evolving pathogens with new agents on the much shorter timescale (rather weeks than years), or powerful new digital technologies (e.g. machine/deep learning, artificial intelligence), and smart computational models, leading to the design and development of the most powerful theranostic options to prevent and treat pandemic–prone diseases in relation to identification of therapeutic targets that can disrupt particular pathogens in the best possible way. Therefore, the eyes of the world should be focused on cutting-edge smart therapies (see Figure 1). It would be advisable if smart therapeutic agents could be affordable, available, easily stored and deliverable to the developing counties too, according to the WHO criteria.

## Short peptides: the most versatile biomolecules

Peptides are smart in nature. They are pre-proteins, associated with RNA, that control and direct all aspects of cellular functions in bio-systems, and by switching receptors and enzymes on and off, coordinate most intercellular communication as perfect bio-messengers (Khavinson et al., 2021). Peptides specificity required for molecular recognition has been refined through evolution over a million years. Short peptides which probably were the catalysts in very early life, have evolved with the human body to have excellent selectivity for specific protein targets (Khavinson et al., 2021). Short peptides combine advantages of small molecules and biologics. They are naturally suited to treating diverse infectious diseases and resistance of micro-organisms to antibiotics, due to their other unique features, such as bio-responsiveness, efficiency, low/no-toxicity, easy design, synthesis and modification, low costs of production, stability under harsh conditions (Apostolopoulos et al., 2021). Peptides effectively inactivate diverse pathogens, regardless of their mutations. They can target viruses at various stages of their life cycle or the host, disrupting protein-protein interactions (Lee et al., 2022). The latter are at the heart of the most important cellular processes and emerging primary targets opening a new era in the pharmacy (Cabri et al., 2021). Notably, there is a plethora of disease-relevant protein-protein interactions, but most of them have been unexplored so far. Proteins of viruses take over cellular host functions through short peptide interaction motifs (in unstructured regions) that bind to defined pockets on globular host domains. These motifs evolve by mutations, enabling viruses to interact with novel host factors (Kruse et al., 2021). An understanding of these peptide-mediated protein-protein interactions could predict viral tropism and molecular processes within host cells (Apostolopoulos et al., 2021).

Peptides are the most versatile motifs offering a much more functional and structural diversity than any other molecules. Recent breakthroughs in diverse fields of science and bio-nano-technological advances have helped to overcome the shortcomings of peptides, such as short half-life, or low bioavailability, to reach their full potential, leading to unlimited bio-applications against pathogens of any origin, developed on a short timescale (Apostolopoulos et al., 2021; Muttenthaler et al., 2021). In consequence, peptides have received renewed attention, and more than 80 peptide-based therapeutics against a wide range of diseases, including viral infections, have reached the global market, and many others (>800) are undergoing (pre)clinical studies (Parra et al., 2022; Wang L et al., 2022) (see Table 1). Peptides targeting protein-protein interactions are of special interest due to the potentially huge impact of PPI in terms of the development of safe and effective drugs where the modality of peptides is key. Three such peptides, nangibotide, reltecimod, and C16G2, against bacterial and viral infections, are in the clinical study currently, and the drug discovery process will evolve soon (Cabri et al., 2021).

**TABLE 1:** Selected peptide-based therapeutics, in clinical use or under clinical studies, for infectious diseases.

Peptide-based therapeutic	Pathogen	Type/Target (Mechanism of action)	Development stage	References
RhACE2-APN01	SARS-CoV-2	hACE2/S protein-hACE2 interaction	Phase- II clinical trial (NCT04335136)	Guo et al. (2021)
Paxlovid (nirmatrelvir and ritonavir)	SARS-CoV-2	main protease inhibitor and antiretroviral protease inhibitor and a strong cytochrome P450	Approved by FDA	Hammond et al. (2022)
Plitidepsin/Solnatide etc. (synthetic peptides)	influenza virus, respiratory syncytial virus (RSV), SARS-CoV (SARS-CoV-2)/SARS-CoV-2	-	III/II	Beheshtirouy et al. (2022)
Canakinumab (anti-inflammatory peptide)	SARS-CoV-2	-	III	(Novartis Pharmaceuticals, (2021); Bagwe et al. (2022))
EpiVacCorona Peptide Antigen-based Vaccine	SARS-CoV-2	Peptide design: SARS-CoV-2 proteins conjugated to a carrier protein	Phase III-IV	(Federal Budgetary Research Institution State Research Center of Virology and Biotechnology “Vector”, (2021); Bagwe et al. (2022))
B-pVAC-SARS-CoV-2	SARS-CoV-2	Peptide design: SARS-CoV-2-derived multi-peptide vaccine	I/II	(University Hospital Tuebingen, (2021); Bagwe et al. (2022))
Multimeric-001 (M-001)	influenza	Composition: Influenza hemagglutinin peptides along with standard vaccine	II	Hamley, (2022)
BIPCV/IMX (V512)	influenza	Composition: Influenza viral peptides	I	Hamley, (2022)
Azatanavir (Reyataz)	Human immunodeficiency virus (HIV)	azapeptide protese inhibitor	-	Deshayes et al. (2022)
Nisin (polycyclic lantibiotic)	antibacterial agent (Gram-positive bacteria)	Depolarization of cell membrane	-	Dijksteel et al. (2021)
Teicoplatin (Targocid) lipoglyco AMP	antibacterial agent (Gram-positive bacteria)	inhibitor of cell-wall synthesis	-	Deshayes et al. (2022)
Polymyxin B (poly-Rx) cyclo-lipo AMP	antibacterial agent (Gram-negative bacteria)	membrane lysin	-	Deshayes et al. (2022)
Anidulafungin (Eraxis; cyclo-lipo AMP)	Antifungal drug	Inhibitor of the beta-(1,3)-D-glucan synthase	-	Deshayes et al. (2022)
Human lactoferrin-derived peptide hLF1-11	Antifungal drug	DNA-binding	I	Magana et al. (2020)

## Landscape of opportunities: topical insights

Currently, more and more research labs around the world are exploring exciting smart therapeutic strategies based on short peptides. It is thus of note that the huge potential of peptides lies in the development of safe and effective either treatment and diagnosis or prevention options. Some of the key issues are discussed briefly. Among the most appealing:

### Modifications and conjugations

Peptide interferons are amyloidogenic peptides that selectively drive aggregation of proteins to inhibit viral/bacterial disease processes. Pept-ins deactivate targeted protein destabilizing pathogens. This is an appealing technology to design broad-spectrum biopharmaceutics, based on the primary sequence, with no information about the protein structure. It is impressive because no protein has been virtually targeted thus far (Michiels et al., 2020; Wu et al., 2021).

Peptide aptamers, known also as peptamers or affimers, are the cornerstone of molecular biology. These chemical antibodies can replace monoclonal antibodies in many applications as a more stable, ethical, sustainable, cheaper, and faster in production, alternative (Kruger et al., 2021). They are small in size (5–20 amino acids residues) and consequently have better penetration of tissues. Peptamers bind to a specific target protein with high affinity and specificity. Thus, they are able to distinguish diverse members from protein family. Furthermore, peptamers can fold into a stable tertiary structure *in vivo,* leading to the higher bioactivity than polypeptides. They are applied as disrupters of protein-protein interactions. They can be effectively used for both *in vitro* and *in vivo* studies. Affimers have unlimited possibilities either in diagnosis or antiviral, antibacterial and antifungal treatment. Diverse modifications, including conjugations can increase a short half-life time of aptamers (Huang et al., 2022).

Peptide nucleic acids (PNA) encode the information of the most important bio-substances of life—proteins and nucleic acids. Thus, they offer huge possibilities in bio-medicine due to their ability to target complementary DNA/RNA strands. Due to the lack of negative charges in terms of peptide backbone excellent bio-chemical stability as well as higher binding energies are the main advantages of PNAs in comparison with DNA/RNA compounds. PNAs are smart scaffolds for breakthrough bio-solutions in diagnosis as imaging agents (Exner et al., 2021). They are promising perfect antisense antibiotics, gene silencers, antivirals, biosensors. They have relevance in genome editing, targeted delivery. Notably, drawbacks of PNAs, such as poor cellular uptake, can be overcome, *inter alia* by combination with nanomaterials, which is based on the control of intermolecular interactions of PNAs with the biological environment. A deeper understanding of theses self-assemblies would be helpful in the further development of PNA-based technology. What is important, PNA-based hydrogels are ideal future multifunctional drug/gene delivery vehicles (Swensen and Heemstra., 2020; Popella et al., 2021; Volpi et al., 2021).

- Nucleopeptides have a great wide-range potential too. The nucleobase-bearing short peptide-based supramolecular structures have natural ability to self-assemble via non-covalent interactions. Supramolecular hydrogels offer specific benefits, such as modularity, tunability, bottom-up design, extracellular matrix-like structure, biomimicry, responsiveness to physical, mechanical, chemical or biological stimuli (Scognamiglio et al., 2021; Giraud et al., 2022). Hence, they are suitable for *inter alia* drug delivery (Noblett et al., 2021). Other innovative applications of nucleopeptides will appear in the next years, due to their appealing features such as reduced cytotoxicity or precise control of the properties (Giraud et al., 2022).

### Antibiotics of the future

Short peptides are emerging broad-spectrum next generation antibiotics. Antimicrobial peptides (AMPs), especially host defense peptides, are first-line pan-agents to combat resistant multi-drug microorganisms, revealing their immense potential and multifaceted nature. AMPs exhibit a unique mechanism of action based on rapid microbes killing at low concentration with immunomodulatory ability and low susceptibility to resistance (Sharma et al., 2022). Interestingly, many AMPs reveal antiviral activity too (Lee et al., 2022). Bomidin, against diverse bacteria and enveloped viruses (SARS-CoV-2, dengue virus, herpes simplex virus, chikungunya virus) can be a good example (Liu et al., 2022). On the other hand, frog-defensin-derived basis peptide can be an effective broad-spectrum agent against influenza and SARS-CoV-2 diverse variants. (Zhao et al., 2022). Antiviral and antifungal mechanisms of AMP action are described elsewhere (Vanzolini et al., 2022). Short and modified AMPs have better therapeutic efficacy, reduced cytotoxicity, decreased proteolytic digestion, cheaper production on large scale (Sharma et al., 2022). These new deep-learning based tools are useful for fast and cost-effective prediction of suitable AMPs (Li et al., 2022). Nanostructured-AMPs have improved stability, lower toxicity and production costs, prolonged activity or controlled delivery (Gera et al., 2022). Interestingly, AMPs found in the secretions of mesenchymal stem cells can attenuate the cytokine storm seen in respiratory diseases. The biopeptides from toxins, ribosomally and post-translationally synthesized peptides, open new ventures for severe respiratory syndrome (corona) viruses (Behsaz et al., 2021). New computational tools and databases are becoming very helpful in prediction and development of new best candidate peptide drugs, reducing costs and time before the *in vitro* experiments (Ramazi et al., 2022; Wang G et al., 2022).

Moreover, macrocycles, able to tackle difficult targets with extended binding sites, should not be neglected. Supramolecular macrocyclic peptides are an appealing platform for the construction of modern bio-materials with remarkable antibacterial activity, as novel antibiotics to combat bacterial resistance (Gao et al., 2021). Glycopeptides, as promising broad-spectrum antimicrobial agents, are worth mentioning too (Dewangan et al., 2021; Acharya et al., 2022).

On the other hand, lipid-conjugated peptides play a role of highly effective viral inhibitors. Nipah virus can be a good example (Marques et al., 2022). Moreover, lipopeptides are useful antigens or adjuvants in the development of vaccines against many infectious diseases. Advantages of lipopeptides include easy design and synthesis with high purity, ability to self-assembly and the potential to adapt specific response (Hamley, 2021).

### Peptide vaccinology

Short peptides are specific antigens and/or adjuvants for modern vaccines. Peptide vaccines have advantages over protein and RNA-based vaccines, overcoming allergic reactions and autoimmune responses. They stimulate durable antibody response (Yang et al., 2022), are safer, relatively inexpensive in large-scale production and highly reproducible, thanks to the latest evolution in solid-phase peptide synthesis, and can be stored for years between outbreaks (Biswas et al., 2022). They can avoid immunopathological pro-inflammatory sequences, off-target antigen loss and mutant escape. They can combine antigens with diverse protective roles or mechanisms, even from different virus proteins (Shalash et al., 2021). Moreover, peptide vaccine sequences may be converted into nucleic acids, and modified to nucleic (or vector-based) vaccines (Jiang et al., 2022). At the beginning of this pandemic, peptide-based vaccines have been unappreciated unfortunately (Shalash et al., 2021). However, a new conjugated self-adjuvanting peptide vaccine with an immune agonist is a promising approach to improve immunogenicity as well as other peptide-based vaccination effects (Long et al., 2022). Synthetic vaccines can be rapidly developed as a fast response against other pandemic-prone pathogens. We can also mention the multi-epitope peptide vaccine against antibiotic resistance (Ismail et al., 2021), or the nanodiamond-peptide-based vaccine as an ‘emergency’ pan-vaccine against newly emerging viruses or bacteria (Billy et al., 2021). Vaccinomics approach and advanced bioinformatics tools are helpful in design of effective vaccines against Marburg (Pervin and Oany, 2021), Nipah (Soltan et al., 2021), Zika (Antonelli et al., 2022), Ebola (Nandy et al., 2018; Mustafa et al., 2021), Lassa (Omoniyi et al., 2021), rift valley fever (Fatima et al., 2022) viruses as well as malaria (Aza-Conde et al., 2021) caused by parasites—potential agents of pandemics, including bioterrorism. The ‘smart’ vaccine strategy uses advanced machine learning to peptide-based epitope mapping and it precisely predicts the binding between viral peptides and human proteins, leading to an increase in the speed of the design and development of broad spectrum (‘universal’) vaccines (Du et al., 2022). This computational approach, thanks to continuous progress in bioinformatics, structural biology (including the huge growth in high-resolution 3D structures of proteins) and genomics, has economic and time-effective value, shortening many experimental steps, and will leading to new advanced paradigms for vaccines design for any upcoming deadly pathogen (Manna et al., 2021; Soltan et al., 2021).

### Peptide nanotechnology and evolving other options

Overall, nano-peptide-based-technology is a great smart innovation for modern bio-medicine. More specifically, triggered transformation of nano-peptides, by interaction with stimuli in bio-environments, facilitates opportunities for the development of smart biomaterials that cannot be achieved with traditional molecules (Nahhas et al., 2021). The flexibility in inter-and intramolecular interactions of short peptides creates space for construction of supramolecular nanostructures with diverse applications. Supramolecular short peptide nanomaterials, used either *in-vitro* or *in vivo*, have antibacterial applications guarantying substantial recognition*,* ease of fabrication, favorable physico-chemical features, biosafety, biocompatibility, biodegrability. These nanomaterials, including peptide composites and biomineralized nanomaterials, also have potential to be antibiotics of the future (Abbas et al., 2022). Peptide nanocarriers reach destination safely and reduce the immune response. Pepsomes are smart liposomes that are responsive to (disease) signals and can be trigger-released at the intended site. They are a prominent strategy for oral delivery carriers of peptide-based drugs (Jash et al., 2021).

A major challenge for novel RNA-based therapeutics is a delivery of drug to the tissue and the cell type of interest. The siRNA-peptide dendrimer nanoparticles achieve safe and efficient siRNA delivery into the lungs, enhance binding properties, viral gene silencing, and antiviral capacity, leaving ‘normal‘ cells unharmed (Khaitov et al., 2021).

Another interesting issue can be self-assembling peptides. They are smart nanoplatforms mimicking viruses through creating their simplified versions by the design of supramolecular bio-materials, where interactions can be precisely manipulated. It offers the opportunity to tackle the challenges of viral infections (Du et al., 2022).

3D bioprinting is worth mentioning too. The cutting-edge biofabrication technology for the automated production of tissue/organ models in the future can control infectious diseases and speed up the construction of complex 3D structures with multiple biomaterials, to prevent the spread of pathogens and understand mechanisms of infections. Ultra short peptide-based hydrogels as bioinks help to shape cells into viable tissues (Das and Das, 2021; Susapto et al., 2021).

To sum up, short peptides and even simple derived amino acids (Bojarska et al., 2021) have huge potential in the treatment of pandemic-prone pathogens, as a broad-spectrum agents.

## Concluding remarks

In the 21st century, infectious outbreaks are inevitable, but not pandemics. Science has tools to save millions of lives. This opinion article has discussed pandemic challenges and enormous potential of short peptides, both synthetic and nature-inspired, that are cost-effective and easy to develop in terms of time and technology, in smart therapeutic strategies for promoting further research in this field. Smart peptide-based approaches are revitalizing the anti-infectious arsenal, and will revolutionize medicine, leading to unlimited therapeutic possibilities.

However, to guarantee successful realization of ambition scientific goals in relation to smart therapies, strong funding support and more holistic anti-pandemic strategies are required. There is still much work to be done to build more advanced global health networks, and trust in pharma to address vaccine hesitancy. More strongly implemented mechanisms of sustainable development and approach to living, from harm to harmony, by protecting biodiversity of our planet, and eliminating warfare as pre-emptive ‘vaccines‘, are needed. Globally, no one is safe until everyone is safe. We must continue global cooperation towards rapid progress on the development of cutting-edge smart therapies, address suitable questions to improve therapeutic options, and consequently prevent and treat viral and bacterial infectious diseases faster than ever before.
